# A study protocol for the development of a SPIRIT extension for trials conducted using cohorts and routinely collected data (SPIRIT-ROUTINE)

**DOI:** 10.12688/hrbopenres.13314.1

**Published:** 2021-07-29

**Authors:** Megan McCarthy, Linda O'Keeffe, Paula R. Williamson, Matthew R. Sydes, Amanda Farrin, Fiona Lugg-Widger, Gwyneth Davies, Kerry Avery, An-Wen Chan, Linda Kwakkenbos, Brett D. Thombs, Alan Watkins, Lars G. Hemkens, Chris Gale, Merrick Zwarenstein, Sinead M. Langan, Lehana Thabane, Edmund Juszczak, David Moher, Patricia M. Kearney

**Affiliations:** 1 School of Public Health, University College Cork, Cork, T12 XF62, Ireland; 2 MRC/NIHR Trials Methodology Research Partnership, Department of Health Data Science, a member of Liverpool Health Partners, University of Liverpool, Liverpool, L69 3BX, UK; 3 MRC Clinical Trials Unit at UCL, University College London, London, WC1V 6LJ, UK; 4 CTRU at Leeds Institute for Clinical Trials Research, University of Leeds, Leeds, LS2 9JT, UK; 5 Centre for Trials Research, Cardiff University, Cardiff, CF14 4YS, UK; 6 UCL Great Ormond Street Institute of Child Health, University College London, London, WC1N 1EH, UK; 7 National Institute for Health Research Bristol Biomedical Research Centre and Bristol Centre for Surgical Research, Bristol Medical School: Population Health Sciences, University of Bristol, Bristol, 1QU BS8, UK; 8 Institute of Health Policy, Management and Evaluation, University of Toronto, Toronto, ON M5T 3M6, Canada; 9 Department of Psychology, Radboud University, Nijmegen, 6525 XZ, The Netherlands; 10 Faculty of Medicine, McGill University, Lady Davis Institute of Medical Research, Jewish General Hospital, Montreal, H3T 1E2, Canada; 11 Swansea University Medical School, Swansea University, Swansea, SA2 8QA, UK; 12 Department of Clinical Research, University of Basel, Basel, Switzerland; 13 Neonatal Medicine, School of Public Health, Imperial College London, Chelsea and Westminster campus, London, SW7 2AZ, UK; 14 Department of Family Medicine, Western University, London, N6G 2M1, UK; 15 Faculty of Epidemiology and Population Health, London School of Hygiene and Tropical Medicine, London, WC1E 7HT, UK; 16 Department of Health Research Methods, Evidence, and Impact, McMaster University, Hamilton, L8S 4K1, Canada; 17 Nottingham Clinical Trials Unit, School of Medicine, University of Nottingham, Nottingham, NG7 2RD, UK; 18 Centre for Journalology, Clinical Epidemiology Program, Ottawa Hospital Research Institute, Ottawa, ON K1H 8L6, Canada

**Keywords:** SPIRIT, routinely collected data, reporting guideline, cohort, electronic health records, electronic patient records, registries, registry-based randomised controlled trial

## Abstract

**Background:** Protocols are an essential document for conducting randomised controlled trials (RCTs). However, the completeness of the information provided is often inadequate. To help improve the content of trial protocols, an international group of stakeholders published the Standard Protocol Items: Recommendations for Interventional Trials (SPIRIT) Initiative in 2013. Presently, there is increasing use of cohorts and routinely collected data (RCD) for RCTs because these data have the potential to improve efficiencies by facilitating recruitment, simplifying, and reducing the cost of data collection. Reporting guidelines have been shown to improve the quality of reporting, but there is currently no specific SPIRIT guidance on protocols for trials conducted using cohorts and RCD. This protocol outlines steps for developing SPIRIT-ROUTINE, which aims to address this gap by extending the SPIRIT guidance to protocols for trials conducted using cohorts and RCD.

**Methods:** The development of the SPIRIT-ROUTINE extension comprises five stages. Stage 1 consists of a project launch and a meeting to finalise the membership of the steering group and scope of the extension. In Stage 2, a rapid review will be performed to identify possible modifications to the original SPIRIT 2013 checklist. Other key reporting guidelines will be reviewed to identify areas where additional items may be needed, such as the Consolidated Standards of Reporting Trials (CONSORT) extension for trials conducted using cohorts and RCD (CONSORT-ROUTINE). Stage 3 will involve an online Delphi exercise, consisting of two rounds and involving key international stakeholders to gather feedback on the preliminary checklist items. In Stage 4, a consensus meeting of the SPIRIT-ROUTINE steering group will finalise the items to include in the extension. Stage 5 will involve the publication preparation and dissemination of the final checklist.

**Conclusion: **The SPIRIT-ROUTINE extension will contribute to improving design of trials using cohorts and RCD and transparency of reporting.

## Introduction

Randomized controlled trials (RCTs), the gold standard for the conduct of clinical research, have revolutionised the way that care is delivered to patients ^1, 2^. However, the frequently high cost of trials, the complex regulatory environment, and delays in incorporating trial findings into clinical practice threaten the ability of trials to continue to improve patient care into the future ^3– 7^.

A protocol is an essential document for any RCT. High quality protocols can assist efficient conduct, reporting, replicability and external review ^8^. However, the completeness of the information provided in trial protocols is often inadequate ^8^. One study carried out by Pildal and colleagues in 2005 found that many trials do not have clear allocation concealment according to their protocol ^9^. In an effort to improve trial reporting quality, an international group of stakeholders launched the Standard Protocol Items: Recommendations for Interventional Trials (SPIRIT) Initiative in 2013 to provide evidence-based recommendations for the minimum set of items that need to be included in trial protocols. The core outputs of SPIRIT are the SPIRIT 2013 Statement, comprising of a 33-item checklist of minimum suggested protocol items with a diagram and an Explanation and Elaboration paper ^10^. The SPIRIT website contains further information and resources ^11^. The SPIRIT guidance has been instrumental in promoting high quality and transparent reporting of evaluations of interventions ^12^. It provides the minimum guidance applicable for all clinical trial interventions and recognises that certain interventions may require extension or expansion of these items ^9, 10, 12^. 

In an observational cohort study, data collected from a group of individuals are gathered for the goal of conducting research ^13^, whereas routinely collected data (RCD) refers to data collected for purposes other than research ^14^. Recently, there is increasing interest and use of cohorts and RCD in RCTs because these data sources have the potential to improve efficiencies by facilitating recruitment, simplifying and reducing the cost of assessment of outcome measures and improving the applicability of trial findings ^15^. Using RCD for RCTs offers novel concepts of testing health care interventions embedded in IT systems used in routine care and fosters conducting large pragmatic trials ^16^. The use of cohorts and RCD provide the potential opportunity for low-cost long-term follow up minimising burden on participants ^17^. To date, only a small number of trials access RCD to inform participant data, and few provide details on the use of such data sources ^18^ but designs for trials conducted using cohorts or RCD including administrative databases, registries, and electronic health records (EHRs) are increasingly used in healthcare evaluation. One study carried out in the UK found that almost fifty percent of all publicly funded trials in the UK intend to collect data from RCD sources ^19^. 

The Strengthening the Reporting of Observational Studies in Epidemiology (STROBE) statement was developed in 2004 to help improve the transparency of reporting of observational research ^20^. In response, the REporting of studies Conducted using Observational Routinely-collected Data (RECORD) statement was developed as an extension to the STROBE statement to help address reporting items of a particular relevance to observational studies using RCD ^14^. While, STROBE and RECORD have been instrumental in improving the transparency of reporting of research, both STROBE and RECORD are only intended to apply to observational research studies ^14^. Thus, the recently developed CONSORT-ROUTINE extension extends the CONSORT guidance to aspects of trial reporting specific to trials conducted using cohorts and RCD data ^21^ while our SPIRIT-ROUTINE extension aims to extend the SPIRIT guidance to protocols for trials conducted using cohorts and RCD. Guidance for what is expected in a protocol using RCD may lead to improvements in accessing data. For example, protocols that contain governance and technical information typically required by a data provider to release their data, will arguably expedite both information governance approvals and data provisioning processes and potentially lead to greater use of RCD ^22, 23^. There is currently no specific SPIRIT guidance on protocols for trials conducted using cohorts and RCD ^5^.

The aim of this project is to outline the steps for the development of the SPIRIT-ROUTINE which will address this gap by extending the SPIRIT guidance for trials conducted using cohorts and RCD.

## Methods

This project is registered with the Enhancing the QUAlity and Transparency Of health Research (EQUATOR) Network since the 5 ^th^ of May 2021 ^24^. The development of this SPIRIT-ROUTINE extension will involve using a sequential approach, following previously published guidance on the development of protocols and the EQUATOR Network methodological framework ^25^, consistent with the recently developed CONSORT Extension for Trials Conducted Using Cohorts and Routinely Collected Data (CONSORT-ROUTINE) ^10^; and similar to other SPIRIT extensions ^12, 26^. The development process involves 5 stages. Stage 1 consists of a project launch meeting to specify and finalise the scope of the extension and definition of cohorts and RCD. In Stage 2, a rapid review will be performed to identify possible modifications or new items to the original SPIRIT checklist. Potential items should have a specific relevance to trials using cohorts or RCD. We will then identify items for modification and places where additional items may be needed by reviewing the CONSORT-ROUTINE statement. Stage 3 will involve an online Delphi exercise, consisting of two rounds and involving key stakeholders to gather feedback on the draft checklist items. Stage 4 will involve a consensus meeting of the SPIRIT-ROUTINE team to suggest any new items and finalise all of the items to include in the extension, followed by stakeholder piloting of the checklist. Stage 5 will involve the publication preparation, dissemination and implementation of the final checklist.

### Stage 1: Project launch and conceptual framework

A project operational team and a study steering committee have been established to deliver the project aims. The project operational team comprises, the lead Principal Investigator, Senior Research Fellow and Research Assistant. The SPIRIT-ROUTINE steering group was established to oversee the conduct and methodology of the project and comprises experts in trial methodology, including members who have conducted trials using cohorts and RCD; experts in the development of reporting guidelines and members of the SPIRIT and Trial Methodology Research Partnership (TMRP) Health Informatics and Outcomes working groups ^27^. All members of the research team will advise on each stage of the project and will help draft and disseminate the final guideline document.

### Stage 2: Rapid review

To create an initial ‘long list’ of items to consider for the SPIRIT-ROUTINE extension reporting guideline checklist, items from SPIRIT 2013 will first be examined to identify where modifications will be needed for trials conducted using cohorts and RCD, and items from the CONSORT-ROUTINE reporting guidelines will be investigated to identify supplementary items to SPIRIT items.

A search of the US National Library of Medicine’s clinical trial registry ( ClinicalTrials.gov) will then be undertaken to find trial protocols using cohorts and RCD in Canada and the US (National Institute of Health (NIH) funded US trials) within the last five years. In addition, a similar search of the National Institutes of Health Research (NIHR) journals library in the UK will be undertaken. Search strategies are available in *Extended data* ^28^.

These institutes operate within different geographies but with broadly similar objectives. In the US, the NIH, is the “nation’s medical research agency and aims to seek essential knowledge about the nature and behaviour of living systems and the application of that knowledge to help improve health and reduce illness and disability” ^29^. The NIHR is the UKs largest funder of health and care research and the NIHR Health Technology Assessment programme contains published trial reports which are of high quality and provide detailed trial information ^30, 31^. 

The results of each search will be individually downloaded into the citation management database Mendeley, and any duplicates will be removed. The eligibility of each protocol will be assessed through a two-stage process. Firstly, one reviewer will screen titles and abstracts. For an article to meet the criteria for full-text review, one reviewer must identify it as potentially eligible. Subsequently, a full-text review will be completed, with two investigators independently reviewing each article. Discrepancies after full-text review will be resolved by consensus, with a third investigator consulted if required. If the number of protocols identified is large, we will select a random sample for review and data extraction.

The eligibility criteria of each publication will be assessed as follows:

*Inclusion criteria*

• RCT of any type
• use of cohorts and RCD; and
• availability of a protocol

Articles which are eligible will be examined to create a long list of items as follows: for trial protocols that describe aspects of methods or reporting of trials conducted using cohorts or RCD, we will examine the protocols and identify areas of trial design that are important to report. Potential items should be applicable to trials using cohorts or RCD and should clarify or alter an existing SPIRIT 2013 item or suggest a new element that should be separately reported as an item. Investigators will check elements for redundancy, and if either investigator or both deem it significant to report, the item will be included in the long list. The items identified will be added to an initial ‘long list’ of items, following the removal of duplicate records. Two investigators will independently extract the data from each protocol using a predesigned data extraction form, available in *Extended data* ^28^. Before starting the Delphi process, we will remove items that are evidently not applicable to trials using cohorts or RCD.

### Stage 3: Delphi exercise

The aim of the Delphi process is to evaluate the list of items for consideration to be included in the SPIRIT-ROUTINE extension and to identify any possible items that may not have been identified in the review. The Delphi survey will consist of two rounds and invitations to participate will be sent out via email for each round. Participants will complete the Delphi survey online using the COMET DelphiManager software ^32^ and all participants will have a maximum of two weeks to complete each survey. The Delphi study participants will include members of the research team, clinical trialists, trial methodologists, guideline experts, Trial Methodology Research Network (TMRN) members and Patient and Public Involvement (PPI) contributors; participants may be added at the rapid review stage (e.g., authors of published trials using cohorts and RCD). While no official guidance on the minimum or perfect panel size for Delphi studies is available, we will aim to include a minimum of twenty participants, as this has been indicated by other studies to deliver reliable results ^5^. 

In round one, we will invite participants to rate items based on how valuable they are for the reporting of trial protocols on a Likert scale of 1–9, which will be labelled as: 1- 3= ‘not critical’ (items should not be part of the SPIRIT-ROUTINE extension checklist), 4–6= ‘no consensus’ (items should be discussed), 7–9 = ‘critical to include’ (item should be part of SPIRIT-ROUTINE extension checklist). All items rated from round one will be brought forward to the second round. In the second round, participants will be displayed the distribution of scores from other participants and the score that they attributed to each outcome, along with any comments from the previous round. They will be asked to reflect, and re-score if they want to, after having been shown the other participants’ views. DelphiManager enable this procedure by an easy setup followed by an inbuilt functionality to calculate the distribution of scores for a certain round ^33^. In comparison to other online survey tools, the score distribution is then automatically presented to the participant in the next round and participants are reminded of their own score. Any item that is rated in the category ‘critical to include’ by more than 75% of the Delphi respondents will be deemed “consensus in”; likewise, if 75% or more score an item as ‘not critical’ it will be deemed ‘consensus out’. Participants will also be able to provide feedback when they rate items and can suggest any additional potential items, along with a rationale. New items will be added to the list for the second round if two or more participants suggest its inclusion, and it is not deemed to duplicate or have any major similarities with any other items already in the survey ^28^. All responses will be anonymous and confidential. Results of the second round of the Delphi exercise will be presented during a consensus meeting to help inform the selection of checklist items.

### Stage 4: Consensus meeting and finalising items for inclusion

A consensus meeting will be held with members of the study operational team, the SPIRIT-ROUTINE steering group and other key stakeholders including, guideline experts, clinical trialists, trial methodologists and PPI contributors. This meeting will be held with approximately 20 participants to establish consensus on the items to be included in the SPIRIT-ROUTINE extension checklist which will include developing item explanations and assessing consistency with the CONSORT-ROUTINE extension. This process will involve presentations of items by individuals with expertise, following a discussion. Firstly, the items in the Delphi survey which reached consensus will be discussed, following any possible objections. Subsequently, outstanding items will then be examined, and meeting participants will be provided with the opportunity to discuss each item. Participants will also be provided with the opportunity to discuss any items excluded during the Delphi process and can propose better explanations of any excluded items; this may involve attempting to alter the team’s conclusion about a particular item. We will arrive at consensus among meeting participants by implementing anonymous voting. Items with 75% or more of voters voting for its inclusion will be retained.

### Stage 5: Dissemination and knowledge translation

Knowledge translation is increasingly recognised to be an ‘ *intensely social process’* that depends on relationships between those who produce research evidence and users of that evidence ^3^. The emerging and existing relationships between the primary investigator and her research team, members of the TMRP and collaborators will be used to disseminate the SPIRIT-ROUTINE extension.

The main outcome of the proposed research will be the development of the SPIRIT-ROUTINE extension for trials which utilise cohorts and RCD. The SPIRIT-ROUTINE extension will contribute to transparent reporting of these trials. The investigators will register their intent to proceed with this exercise with the EQUATOR network, and our proposed extension will be listed under “Reporting Guidelines Under Development” ^24^. EQUATOR is an “umbrella organization that brings together researchers, journal editors, peer reviewers, research funders, and other collaborators with a mutual interest in improving the quality of research” ^34^. We will use knowledge translation strategies consistent with previously successful efforts by SPIRIT and EQUATOR and members of our team, the majority of whom have such experience.

Strategies for knowledge translation may include:

(1) Involvement of key stakeholders including trialists, trial funders, trial methodologists, guideline experts, regulators, TMRN and Trial Methodology Research Partnership (TMRP), ethics board members, and PPI contributors in the team that is developing the extension with an extended group being recruited for the Delphi process including national and international experts

(2) Publication of the SPIRIT-ROUTINE extension in journals

(3) Dissemination via the SPIRIT group and EQUATOR network, including publication on their websites

(4) Presentations at conferences (e.g. submission to ICTMC 2022) and focused workshops on trials embedded in existing data sources

(5) Dissemination via the TMRN and TMRP with delivery of a Clinical Research Facility-Cork (CRF C/TMRN) webinar on the process of the development of a SPIRIT extension

(6) Dissemination will include presentation at the HRB-TMRN webinar and through relevant social media channels such as Twitter and YouTube

### Ethics statement

The proposed Delphi study abides by the ethical requirements of University College Cork aiming to assure rigour and responsibility in the conduct of research. Ethical approval for the Delphi survey will be sought at a later date from the Social Research Ethics Committee in University College Cork. All Delphi participants identified will be invited to complete each Delphi round via email. Informed consent will be assumed from Delphi participants who complete the survey. Participants will be made aware of the objectives of the Delphi survey in the email and that by completing the survey they will be giving permission for their anonymised responses to be used during the Delphi process, and to be accessed by members of the research team. Participants will also be made aware that their names will not be linked with the research materials, and will not be identifiable during the Delphi survey or in the reports that result from the research.

### Study status

Stage 2, which involves performing a rapid review, has commenced.

## Conclusions

This SPIRIT-ROUTINE extension for trials conducted using cohorts and RCD aims to promote transparency and clarity and to reduce research waste due to inadequate reporting. Consistent with the recently developed CONSORT extension for trials conducted using cohorts and RCD ^5^, this SPIRIT extension is being carried out with the long-term goal of improving the quality of reporting by establishing standards early in the process of uptake of these trial designs. 

## Data availability

### Underlying data

No underlying data are associated with this article.

### Extended data

Open Science Framework: The development of a SPIRIT extension for trials conducted using cohorts and routinely collected data (SPIRIT-ROUTINE). https://doi.org/10.17605/OSF.IO/VQRUW ^28^. 

This project contains the following extended data:

• Appendix 1. SPIRIT-ROUTINE Electronic Search Strategies.docx (search strategy for Stage 2).
• Appendix 2. SPIRIT-ROUTINE Data extraction form.doc (data extraction form for protocols included in Stage 2).

Data are available under the terms of the Creative Commons Attribution 4.0 International license (CC-BY 4.0).
