# Supplementary material for: A study protocol for the development of a SPIRIT extension for trials conducted using cohorts and routinely collected data (SPIRIT-ROUTINE)
Source: HRB Open Res. 2021 Jul 29;4:82. [Version 1] doi: 10.12688/hrbopenres.13314.1 (PMC8609390; doi:10.12688/hrbopenres.13314.1)
Supplement: Peer review history

# Peer review: Reviewer response for version 1

**DOI:** 10.21956/hrbopenres.14495.r30657

**Published:** 2021-11-22

**Authors:** Jim Lewsey

**Affiliations:** 1 Institute of Health & Wellbeing, University of Glasgow, Glasgow, UK

**Stage 1**

 This stage involves specifying/finalising the definition of cohorts and RCD. It is not clear from the article how this will be achieved. I expect the definition of ‘cohorts’ will be straightforward but routinely collected data may be more difficult?

**Stage 2**

 The data extraction form does not have anything specific relating to cohorts of RCD?

**Stage 3**

 It would be useful to add a sentence or two on what kind of input / engagement you will be expecting from PPI contributors?

**Minor:**

- Typo – p4; “…by extending SPIRIT-ROUTINE…”, ‘the’ not needed.
- Typo – p4; “…involve publication…”, ‘the’ not needed.
- Typo – p5; “1- 3” should be “1–3” to be consistent with other text.
- Typo – p5; “DelphiManager enable this…”, missing ‘s’.
- Typo – p6; The list of strategies has inconsistent indentation.

Is the study design appropriate for the research question?

Yes

Is the rationale for, and objectives of, the study clearly described?

Yes

Are sufficient details of the methods provided to allow replication by others?

Yes

Are the datasets clearly presented in a useable and accessible format?

Not applicable

Reviewer Expertise:

Medical Statistics

I confirm that I have read this submission and believe that I have an appropriate level of expertise to confirm that it is of an acceptable scientific standard.

# Peer review: Reviewer response for version 1

**DOI:** 10.21956/hrbopenres.14495.r30584

**Published:** 2021-11-15

**Authors:** Sallie-Anne Pearson

**Affiliations:** 1 Centre for Big Data Research in Health, Faculty of Medicine, University of New South Wales, Sydney, NSW, Australia

Thank you for the opportunity to review this study protocol relating to the SPIRIT extension for trials leveraging cohorts and routinely collected data.

 I enjoyed reading this paper. It is very timely and bolsters transparency and replicability in both trials and the observational world. I have extensive expertise in the latter, so my comments come from that perspective. I hope the following comments are of benefit to the development of the standardised reporting tool.

 First, a small but important point from where I sit. I feel the paper is confusing issues of design with the nature of data collections. Specifically, I found the reference to cohorts and RCD a bit jarring. Cohort studies sit squarely in the observational world and can leverage both primary and secondary (or routinely collected data). So, I feel it is important for the authors to be a bit clearer about what they mean by cohorts and RCD. Do they mean that the trials may leverage data from existing cohort studies (for example longitudinal cohorts that often leverage both primary and secondary data) and also tap into routine collections? Or something else? This point is rather nuanced but, for someone in my world, quite important.

 Again, a small point – last sentence para 3 page 4 – 50% of trials intend to collect data from RCD sources. RCD data are not collected, rather accessed or linked.

 The methodology to develop the tool is sound and aligns with best practice. I do however have a couple of comments about composition of the steering group and stakeholders providing input. It appears that experts in the ethics, governance, linkage and analyses of RCD do not form part of the steering group. If there is already representation, I strongly suggest this is stated overtly. If not, I believe people with this expertise could add significant value and insights to the process.

 Stage 2. Why is the search limited to trials undertaken in the US and Canada? There are large scale trials linking data in other parts of the globe. Given the jurisdiction specific issues that may need to be addressed, I would have thought a broader search may beneficial. For example, Neal *et al.* (2021) ^1^ is a great example of linkage to RCD in China.

 Stage 3: Again, I would strongly support engaging routine data experts in this phase. They are likely to offer important insights. There are networks such as the International Population Data Linkage Network that could be an appropriate vehicle here. I also note that organisations such as the International Society of Pharmacoepidemiology have also developed statements and guidance that may be useful to link into such as Pratt *et al.* (2019) ^2^.

 I wish the SPIRIT-ROUTINE Steering Group all the very best and look forward to seeing the outcomes of this very important work.

Is the study design appropriate for the research question?

Yes

Is the rationale for, and objectives of, the study clearly described?

Yes

Are sufficient details of the methods provided to allow replication by others?

Yes

Are the datasets clearly presented in a useable and accessible format?

Not applicable

Reviewer Expertise:

I am an expertise in the analysis of routinely collected data to examine the use and outcomes of prescribed medicines use in routine clinical care. I am also a leading authority on the ethics and governance of routinely collected data for population-based research.

I confirm that I have read this submission and believe that I have an appropriate level of expertise to confirm that it is of an acceptable scientific standard.

## References

[1] : Effect of Salt Substitution on Cardiovascular Events and Death. *New England Journal of Medicine* .2021;385(12) : 10.1056/NEJMoa2105675 1067-1077 10.1056/NEJMoa2105675 34459569

[2] : Data linkage in pharmacoepidemiology: A call for rigorous evaluation and reporting. *Pharmacoepidemiol Drug Saf* .29(1) : 10.1002/pds.4924 9-17 10.1002/pds.4924 31736248
